# Genetic Improvement in Dromedary Camels: Challenges and Opportunities

**DOI:** 10.3389/fgene.2019.00167

**Published:** 2019-03-12

**Authors:** Mohammed A. Al Abri, Bernard Faye

**Affiliations:** ^1^Department of Animal and Veterinary Sciences, Sultan Qaboos University, Muscat, Oman; ^2^UMR SELMET, CIRAD-ES, Montpellier, France

**Keywords:** dromedary camels, genetic improvement, challenges, opportunities, food security, climate change

## Introduction

Adaptation to a hotter climate is vital for future livestock as heat stress can extremely reduce their productivity, health, and fertility (Hayes et al., 2013). Camels have developed, through millennia, the ability to produce quality meat, milk, and fiber in some of the hottest and most hostile environments in the globe. According to the FAO live animals statistics, the worldwide camel population is ~35 million heads (FAO, 2019), most of which are in Somalia, Sudan, Niger, Kenya, Chad, Ethiopia, Mali, Mauritania, and Pakistan. Moreover, partly due to climatic changes, areas of camel rearing are expanding, especially in Africa (Faye et al., 2012). Among the large camelids (dromedary and Bactrian), dromedary camels compose about 95% of the population (Bornstein and Younan, 2013). Due to their unique physiology and in light of the current climate change impacts on ecosystems, camels are poised to be an excellent candidate species for production (Hoffmann, 2010). This is specifically true in regions where agro-pastoralism is being replaced by pastoralism due to climate change (Bornstein and Younan, 2013). However, to harness their potential, an improved understanding of the genetics underlying their unique biology is needed.

## Opportunities

The term “Livestock Revolution” was coined to describe the projected increase in demand for animal products due to population growth, increased income, and urbanization in developing countries. For example, demand for beef and milk is expected to rise to 2.7–30 million metric tons, respectively by the year 2020 (Delgado et al., 1999). Most camels are in developing countries and can contribute in meeting meat and milk demands if utilized efficiently. Currently, most of that demand in many Middle East and North Africa (MENA) countries is met either by importation or local production using commercial exotic livestock not adapted neither to local climatic conditions and low input systems dominating the region. Camels can not only contribute in boosting food security but also in job creation, poverty alleviation and economic diversification. Utilization of camels in production will also reduce their destructive impact on the environment as is the situation in Australia (Saalfeld and Edwards, 2010). There, camels have contributed to the reduction of vegetation not only due to the increase in sheer numbers but also because they can browse and graze on a wide range of plants that are avoided by or are inaccessible to other livestock such as thorny bushes (Stiles, 1988; Faye, 2011; Al-Jassim and Sejian, 2015).

Beside their adaptation to harsh environments, camels are multipurpose animals used for milk and meat production, hair/felt, racing, transportation, and tourism. Camels also have a slow metabolism which results in comparatively less feed requirements compared to other ruminant livestock. As a result, they produce less methane on the basis of body mass index (Dittmann et al., 2014). Moreover, camels' milk and meat are highly nutritional and are comparable and sometimes deemed better than cattle beef and milk. For instance, camel meat contains less fat than lamb or beef (Kadim et al., 2008) and its protein quality, assessed by the index of essential amino-acids in meat, is the highest among red meat (Raiymbek et al., 2015). Its milk contains between 3 and 10 times more vitamin C than cows' milk (Faye et al., 1997; Konuspayeva et al., 2009). It also contains lower β-casein and no β-lactoglobulin resulting in its hypo-allergic property (Konuspayeva et al., 2009). During the last decade, demand for camel milk and meat products have increased both locally (in arid regions) and internationally with products varying from milk and its derivatives to beauty products to hump fat. Thus, a number of camel intensive dairy farms have been established worldwide and are currently supplying local and international markets (Gossner et al., 2014). There is, therefore, a slow but steady integration of camels' products into national and global economies (Faye, 2018a).

However, to utilize camels' potential, they need to undergo genetic improvement while sustaining their genetic diversity. Examples of successful genetic improvement of production traits in other livestock species are plenty and have considerably reshaped the livestock industry worldwide. For example, pigs are now 25% leaner and grow faster today than 20 years ago (Rothschild and Plastow, 2008) and milk production of Holstein Friesian cattle saw an increase of 40–80 kg/cow/year between 1980 and 2010 (Hayes et al., 2013). Similar success stories are evident in poultry and beef cattle and, together, have resulted in cheaper and more abundant animal derived proteins being available to consumers. Through multi-trait genetic improvement programs, not only production traits can be improved, but also health traits such as resistance to Peste des petits ruminants (PPR) virus or Rift Valley fever (RVF) both of which can have devastating effects on camel health. In addition, genetic improvement can also target other commercially important traits such as racing ability, beauty (Faye, 2015) or ease/suitability for machine milking (Ayadi et al., 2013).

Relatively few studies have investigated the genetic variability of production traits in camels (Dioli, 2016; Hemati et al., 2017). However, the few studies that have been carried out so far indicated that camels have a high genetic variability which is due to the lack of selection and the current and historical movements of camels between countries for trade and sometimes war (Almathen et al., 2016). This variability was reflected in the heritabilities of various traits, indicative of the potential for ample genetic gain if systematic selection is to be implemented. For instance, heritability estimates of body weight and growth rates were moderate to high, 0.24–0.40, respectively (Al-Sobayil et al., 2006). In another study, heritability estimates for birth weight was 0.37 and that of average daily gain ranged between 0.25 and 0.49 (Almutairi et al., 2010). Also, the heritability estimates for milk yield at 305 days and test day yields were 0.24 and 0.22, respectively (Almutairi et al., 2010). Together, these heritabilities show that the respective traits can indeed be improved through selection.

Genetic improvement in camels can be pursued using various methods. The first is single gene tests currently incorporated into selection programs of other livestock (Rothschild, 2004). However, to our knowledge, apart from color coat genes (Almathen et al., 2018), no other traits have been mapped in camels in which single test genes can be developed for. The second is traditional genetic selection using Best Linear Unbiased Prediction (BLUP) to estimate Estimated Breeding Values (EBVs) using phenotypic and pedigree information (Henderson, 1984). A variation of this method is using genomic relationships (using molecular markers) instead of pedigree information (Rodríguez-Ramilo et al., 2015). The third is using Genomic Selection (GS) which calculates Genomic EBVs termed “GEBVs” (Meuwissen et al., 2001). GEBVs are calculated as the sum of the effects of genetic markers across the entire genome of each animal (Hayes et al., 2009). This method requires that the genetic marker effects be inferred from individual single nucleotide polymorphism (SNPs) on a large reference population with phenotypic information. Once these effects have been calculated, only marker information is required to calculate GEBV in later generations (Hayes et al., 2009). GS or BLUP using genomic relationships are thus most likely to be adopted in camel genetic improvement especially because camels are not traditionally pedigreed. GS is specifically recommended for camels due to their long generation intervals and can accelerate the rate of genetic gain compared to conventional selection schemes. Unlike in small ruminants where the generation interval is short and a cost benefit analysis has to justify the implementation of GS (Mrode et al., 2018), in large ruminants such as camels and cattle, the high benefits of GS are clear with higher genetic gains and profits as a result of the reduction in generation intervals (Konig et al., 2009). Additionally, GS can result in increased accuracies of EBVs for young bulls and reduces the cost of progeny testing. In later generations, when more pedigree and phenotypic data become available, GS can be combined with individual and progeny phenotypic information in selection schemes. Moreover, accurate parentage testing can be obtained as a byproduct of genotyping animals for GS. A limitation in implementing GS however is the cost of genotyping, although that can be mitigated by using low density SNP panels (Abo-Ismail et al., 2018) or genotyping only a fraction of the genome, using Restriction-associated DNA (RAD) sequencing (Kess et al., 2016) or Genotyping by sequencing (GBS) (Elshire et al., 2011).

There is indeed an immediate potential in the existing camel dairies worldwide to ignite the spark of camel genetic improvement as they are consistent in pedigree and phenotypic collection. The different farms in Saudi Arabia (SA), United Arab Emirates (UAE), Kenya, and Bahrain can be the starting point for a genetic improvement in dairy camels if they participate in a common genetic evaluation program. To our knowledge, little communication and collaboration is currently practiced between these dairies, due primarily to competition. However, this lack of collaboration is bound to fade away with the realization that cooperation will improve long term profitability. Under such cooperation, records pertaining to milk production and health traits can be exchanged between the dairies as well as verified pedigree data. This exchange can help create a virtually common nuclear flock which can be utilized for traditional genetic evaluation of sires and dams. At a later stage, genomic selection can be practiced in order to speed up the genetic gain. In addition, genetics of the elite animals can be disseminated to camel owners in respective countries. The realized genetic gain in the camel owners' herds shall encourage them to participate in genetic improvement programs. This will increase the number of participatory herds and the genetic variability accessible to the genetic evaluation program and accelerate genetic improvement. As the numbers of herds increase and more pedigree data become available, the evaluation can be extended to other traits such as beef, racing, and beauty. That in turn will help with the classification of the camel population into beef and dairy individuals and the identification of elite individuals in each category. This classification and will later make it easier for investors and owners to make future breeding decisions and reinforce the industry. An alternative to starting with the camel dairies for genetic improvement is starting with the camel owners themselves by forming cooperative community based breeding programs. These can begin with a nuclear flock formed by the owners that expand to include more owners in future. Such programs are found in developing countries for sheep and goats (Wurzinger et al., 2011) and have been successfully implemented in small ruminants (Gizaw et al., 2014; Mrode et al., 2018). This is, however, a more challenging approach and requires more upfront investment mostly by the funding agencies. In order to reduce the running cost, this approach needs to make use of modern digital systems such as mobile phones or tablets for recording performance and pedigree data (Mrode et al., 2016) and perhaps novel technologies such as automated monitoring systems which are now successfully used in dairy cattle (Stangaferro et al., 2016).

## Challenges

Despite its unique potential and increased contribution to food security, comparatively less attention has been paid to camels compared to other livestock species (Faye, 2015). Camels' genetics and genomics research is not an exception to this trend. Consequently, there are relatively few published studies in the area of camel genetics and genomics albeit ongoing research efforts (Jirimutu et al., 2012; Burger and Palmieri, 2014; Al-Swailem et al., 2018) notably through the International Camel Consortium for Genetic Improvement and Conservation (ICC-GIC) initiative. This is due, in part, to the lack of genomics tools to conduct such studies. For instance, the camel reference genome has not yet been released and no commercial genotyping platform has been developed for the species. Such platforms can be used to discover QTLs with impacts on specific traits using Genome Wide Association Studies (GWAS) and are the main engine for GS programs. Thus, whilst many QTLs have been reported using GWAS in sheep, cattle, and horses, none have been reported in camels. For example, endurance racing in Arabian horses was found to be partially controlled by 5 QTLs (Ricard et al., 2017) while in thoroughbred racing horses, a single mutation in the myostatin gene (*MSTN*) was found to profoundly affect the racing speed and stamina (Bower et al., 2012). Also, in cattle, variations in the *FABP4* gene were found to be significantly associated with milk yield and milk protein percentage (Zhou et al., 2015). Additionally, with the exception of dairy camels and to a less extent in racing in Dubai, very limited traditional genetic selection is applied (Faye, 2015).

Moreover, countries harboring most of the camel population are in different development stages pertaining to agriculture and infrastructure development. Thus, creation of intensive or peri-urban camel dairy or beef industries requires immense infrastructure investments, support and coordination between all stakeholders all of which are challenging. Although there is a gradual urbanization of some of the pastoral camel populations (Faye, 2015), most of the camel populations are still under traditional farming systems. As a result most camels do not possess unique identification number which hampers pedigree recording, good farm management, and performance recording (Caja et al., 2013). The relatively small herd size and scattered herds further complicate this issue making it difficult and costly to collect phenotypic data.

Another challenge facing the genetic improvement in camels is difficulty in disseminating superior genetics due to the difficulty of performing Artificial Insemination (AI). This is due primarily to the difficulty in semen collection and handling (due to the gelatinous nature of seminal plasma). In addition, deep freezing of camel semen has proved to be highly a challenge. Although research groups have tried different buffers and diluents as mediums for freezing camel semen (Skidmore et al., 2013), to date, it remains a challenge facing AI in camels. Moreover, unlike cattle, female camels are induced ovulators i.e., the females need to be induced to ovulate prior to AI (El-Bahrawy, 2018). While it is possible to use GnRH for inducing ovulation in camels, it depends on the stage of follicular development and/or estrous cycle (Manjunatha et al., 2015). A promising protocol for timed breeding called FWsynch in which a GnRH and PGF2α based hormonal regimen to synchronize the follicular wave was recently developed with satisfactory results (Manjunatha et al., 2015). However, while the cost of implementing such timed breeding regimens can be justifiable by research centers and camel dairies, they may not be as such for many camel owners specially that most of them reside in remote areas.

In the era of genomics, phenotypes are still very important and the availability of accurate and well defined phenotypes to be used in genetic studies and evaluation programs is imperative (Gonzalez-Recio et al., 2014). Unlike in developed countries, most of the camel herds in developing countries lack breed societies. They also do not have on farm automated milk recording systems and do not collect health or fertility traits (Faye, 2018b). Therefore, phenotypic recording is seldom practiced in camel populations except in intensive dairy farms, research, or racing. This creates an obstacle for genetic improvement programs and would require a serious collaboration of owners and stakeholders to circumvent. If camel breeds are sometimes described at a national level, as for example in Saudi Arabia (Abdallah and Faye, 2012), Tunisia (Chniter et al., 2018), or Algeria (Oulad-Belkhir et al., 2013), there is no standardization of the traits and parameters to be systematically recorded. For example, despite proposal on linear scoring for udder morphology, there is no application at a large-scale recording system (Ayadi et al., 2016).

The final hurdle is that, in developing countries, camels' meat and milk products are generally more expensive than imported milk and beef or those produced locally (by advanced genetic stock from developed countries). This is expected given the cost of production and the lack of genetic improvement in camels. It is therefore challenging for small scale producers to survive without government subsidies and support. To increase the market share and potential for such producers, added value products (such as flavored milk, dry milk, cheese, sour milk, camel burgers, and sausages) need to be produced and smart marketing strategies need to be adopted. Such strategies could include awareness campaigns of the health benefits of camel products, attractive product packaging, online marketing and partnership with existing cattle dairies and beef production firms for distribution and marketing. Camel milk can be marketed as a functional food, optimal for infants and elderly (Nikkhah, 2011). Focus can be made on the antimicrobial, antioxidants, and antidiabetic components of camel milk (Hailu et al., 2016). All of this can increase the value of camel products and hence improve producers' profitability and alleviate their dependence on the governments in the long term. If producers' profitability improved, it would become more feasible for them to participate in genetic selection programs. Selection for economically important traits can be practiced and would reduce the production cost, thereby reducing prices and increasing long term competitiveness.

In conclusion, camels have a large potential that is underutilized due to technical, logistic, political, and economic challenges. However, these challenges are not insurmountable, and much can be done to exploit the camels' potential. Genetic improvement is certainly promising in camels but would require the collaboration of all stakeholders and deeper understanding of the potential of this exceptional animal.

## Author Contributions

MA conceived the idea and wrote the manuscript. BF participated in writing and reviewing the manuscript.

### Conflict of Interest Statement

The authors declare that the research was conducted in the absence of any commercial or financial relationships that could be construed as a potential conflict of interest.
